# Acellular dermal matrix in breast augmentation surgery: A systematic review

**DOI:** 10.1016/j.jpra.2024.02.004

**Published:** 2024-02-15

**Authors:** Caterina Marra, Roberto Cuomo, Alessandra Ceccaroni, Paola Pentangelo, Carmine Alfano

**Affiliations:** a Plastic Surgery Unit, Department of Medicine, Surgery and Dentistry, University of Salerno, Baronissi, 84081, Salerno, Italy; b Plastic Surgery Unit, Department of Medicine, Surgery and Neuroscience, University of Siena, Siena, Italy

**Keywords:** Breast augmentation, Acellular dermal matrix

## Abstract

**Background:**

The use of acellular dermal matrix (ADM) in breast reconstruction was described for the first time in 2000s. Recently, ADMs have been used not only in reconstructive surgery but also in cosmetic breast surgery for both primary and revision indications. Therefore, the matrices represent an important support to recent surgical techniques for breast augmentation in treatment or prevention of complications. Conversely, ADMs can affect operative times, costs, and additional complications related to their placement. A review of the literature was carried out to evaluate the efficacy, safety, and indication for the use of these matrices in cosmetic breast surgery.

**Methods:**

A literature review was conducted including manuscripts published up to April 2023 on breast augmentation using ADM. PubMed and MEDLINE were the databases used for research. The keywords used were “Breast augmentation” and “Acellular Dermal Matrix.” Non-English language articles have been excluded.

**Results:**

The initial search for “breast augmentation” yielded 7900 results, which were further reviewed for “Acellular Dermal Matrices” in breast augmentation, selecting 74 articles. Following further screening, 12 articles were included in the review. A total of 787 patients were treated with breast augmentation and ADM placement. The main indication was capsular contracture (60%).

**Conclusions:**

The current evidence from the published scientific literature, albeit limited, suggests the indication for the use of ADM in revision surgery, to support the prosthetic pocket, to minimize the risk of capsular contracture and its recurrence.

## Introduction

Acellular dermal matrices (ADMs) were first described for reconstructive breast surgery in the early 2000s.^1^ The application of ADMs in postmastectomy breast reconstruction has been well described in literature, however, its use in cosmetic breast surgery is poorly investigated.^2^ With increasing experience by plastic surgeons with ADM in breast reconstruction, its use is moving from purely reconstructive procedures into cosmetic applications in both revision and primary aesthetic surgical practice. Complications in cosmetic breast surgery, especially augmentation, are common, as are revision procedures. With an envelope of thin scar tissue, reoperation can present additional technical challenges and have a significantly higher complication rate than primary procedures.^3^^,^^4^ Most recently, ADM has emerged as a new therapeutic adjunct, in addition to all conventional techniques, for the treatment and prevention of complications, such as capsular contracture, implant malposition, implant wrinkling/rippling, and ptosis, to improve revisionary surgery outcomes.^4^, ^5^, ^6^, ^7^, ^8^ In revision cosmetic surgery, ADMs are employed to provide soft tissue reinforcement, which is often lacking in augmentation patients, provide a means for reinforcing breast pockets for correction of implant malposition, and strengthen thinned soft tissue for the correction of rippling.^9^ However, in addition to the revisions, matrix has also been used in primary mastopexy or reduction mastopexy to provide dermal support to improve breast shape, projection, and to prevent “bottoming-out”.^10^^,^^11^

To date, the use of ADMs has been relatively more limited for cosmetic breast procedures, probably due in part to direct product cost and the significant additional operating time required.^12^^,^^13^

The aim of our study is to review the published literature to date to evaluate efficacy and safety of ADM in cosmetic breast surgery.

## Materials and Methods

We conducted a systematic review according to Preferred Reporting Items for Systematic Reviews and Meta-Analyses guidelines. In this review, we considered the studies published as full-text articles that investigated the use of ADM in breast augmentation. Only articles written in English were included. No publication data limits were set. Surgical technique reports, expert opinions, letters to the editor, studies on animals, unpublished reports, cadaver or in vitro investigations, reviews of the literature, and book chapters were excluded from the present review. Scopus, Cochrane Library, and MEDLINE via PubMed were searched using the keywords: “Breast Augmentation” and “Acellular Dermal Matrix.” Two independent reviewers (A.C. and C.M.) collected the data from included studies. Any discordances were solved by a third author (C.A.). For each study included in the present article, the following data were extracted: primary, secondary surgery or revision surgery, prepectoral or subpectoral breast surgery, indication for use of ADM, number of patients, complications, and mean follow-up (Table 1).

**Table 1:** Flow diagram of studies identified in the systematic review (PRISMA template [Preferred Reporting Item for Systematic Reviews and Meta-Analyses]).

## Results

After screening 7900 results, 74 were considered eligible for full-text analysis. In total, 62 studies were excluded because they did not fit the inclusion criteria. Finally, 12 articles that met inclusion criteria were included in this review. Overall, 787 patients had a breast augmentation with the use of ADM. A total of 484 patients had a breast augmentation with position of ADM in a subpectoral plane, whereas 199 patients had it in a subglandular plane, but those data were not available in all articles (683/787 patients). The mean follow-up was 2.2 years. The indication for the use of ADM was capsular contraction in 472 patients (60%) while 55 patients had a primary breast augmentation. The recurrence rate for capsular contraction after the use of ADM was 1.47% (Table 2).

**Table 2:** Characteristics of the included studies.

Article	Number of patients	Indication	Surgical plane	Follow-up	Complications
1. G. Patrik Maxwell et al. 2009	78 patients	CC 58; Implant exposure 2; Rippling 7; Implant malposition 5; Bottoming out 4; Symmastia 4	56 patients subpectoral 22 patients sub-glandular	1 Year	Recurrence rate 0% Complication: 5.1 % hematoma 1 patient Seroma 2 patients, Malposition 1 patient
2. G. Patrik Maxwell et al. 2013	197 patients	All revision Surgery: CC 115; Implant malposition 58; Rippling 9; Ptosis 7; Implant exposure 3	139 patients subpectoral 47 patients sub-glandular	3.1 Years	Recurrence rate 1,6% Complication rate: 4.8%: - CC 3 patients - Infection 3 - Implant malposition 1 patient - Hematoma 1 patient - Seroma 1 patient
3. G. Patrick Maxwell et al. 2014	106 patients	All revision Surgery: CC 55; Implant malposition 41; Rippling 1; Implant exposure 1; Ptosis 9	Subpectoral 79 Sub-glandular 27	3.1 Years	Recurrence rate 0% Complications: 0.9% (1 infection)
4. Scott L. Spear et al. 2013	Total 39 patients	3 (primary surgery) 33 (revision surgery): Inferior pole support 39; Fold malposition 28; CC 25; Rippling 6; Symmastia 11	8 patients subpectoral 31 patients sub-glandular	1.5 Years	Complications: 2 patients malposition, 1 patient bottoming out, 1 patient infection
5. Tristan L. Hartzel et al. 2010	23 patients	CC 6; Implant malposition 14	All sub-pectoral	2.1 Years	Recurrence rate 10,5% Persistent surface irregularities 4 breasts; complication rate 2,6% (infection 1 breast)
6. Pozner et al. 2013	93 patients	All revision surgery: CC 36; Bottoming out 22; Malposition 16; Ptosis 8; Rippling 7; Symmastia 2; Other 2		1.5 Years	Complication: 1.6% (Extrusion 1; Infection 1)
7. David A. Hidalgo et al. 2020	32 patients	All revision surgery for CC	30 patients subpectoral 2 patients sub-glandular	2.4 Years	Complication: 1 failure for therapy for lung cancer
8. Douglas S. Wagner et al. 2019	43 patients	All revision surgery for CC	21 patients subpectoral 22 patients sub-glandular	4.7 Years	Complications 2.7 % (2 Hematoma; CC 2)
9. C. Bojanic et al. 2021	1 patient	Revision for CC	1 patient sub-glandular position	3 Years	Recurrence rate 0%
10. A. Kornstein 2013	3 patients (Primary augmentation mammoplasty)	All for poor quality soft tissue mammary support	All patients subpectoral	1.5 Years	Recurrence rate 0%
11. G. Patrick Maxwell et al. 2011	78 patients	56 CC; 2 Implant exposure; 7 Rippling; 5 Implant malposition; 4 Bottoming out; 4 Symmastia	56 patients subpectoral 22 patients sub-glandular	1.8 Years	2 complications: Hematoma 1 patient Implant malposition 1 patient
12. T. Roderick Hester et al. 2012	94 patients	45 Prevention or treatment of CC 49 Primary augmentation mammoplasty	69 patients subpectoral 25 patients changed plane from submuscular to sub-glandular	1.3 Years	5 complications in 5 patients (6.25% overall complication rate): - 2 seroma - 3 CC

## Discussion

Breast reconstruction stands out as the most frequently conducted aesthetic procedure globally. Advancements in surgical technique and implant technology have been made but undesired outcomes and complications are encountered.^3^ With the advent of ADM, surgeons can utilize advanced technology and not work only on native tissue. Initially, application of ADMs has been popularized in breast and abdominal wall reconstructions.^9^^,^^14^, ^15^, ^16^, ^17^, ^18^, ^19^ The use of ADM in breast reconstruction is meant to replace tissue, provide an increased area of coverage in the inferior pole, an increased intraoperative fill volume, a better definition of the inframammary fold, reduce device palpability, and favor an immediate implant-based reconstruction, giving the patient an instant positive psychological advantage. Furthermore, capsular contracture is a common complication in breast augmentation, based on our review and literature analysis, the application of ADM reduced capsular contracture rates.^11^^,^^12^^,^^20^, ^21^, ^22^, ^23^ Despite these advantages, recent literature shows an increased rate of complications associated with the use of ADM: seromas, infections, red breast syndrome, and failure of vascularization are reported. ADMs have been investigated mainly in breast reconstruction. However recently, surgeons have started to use it in cosmetic breast surgery for primary and secondary procedures but the evidence in literature about it is poor.^6^^,^^7^^,^^24^, ^25^, ^26^

Breast augmentation is one of the most demanded procedures in plastic surgery and one of the most commonly performed by plastic surgeons.^27^ Breast augmentation and augmentation/mastopexy are associated with a remarkable primary and secondary revision rate. Approximately, 15%–30% of primary augmentation patients undergo revision within 3–6 years from the first surgery, and approximately 30%–40% of revision augmentation patients undergo further revision within 6 years. Capsular contracture (CC), rippling, implant malposition, ptosis, and asymmetry are some of the main reasons for revision. These patients have a thin and scarred tissue envelope and reoperation procedure can present various technical difficulties.^4^^,^^28^ CC is a common complication of breast augmentation often requiring revision surgery. There are many techniques for prevention and treatment. Capsulotomy or total capsulectomy, along with implant removal and pocket change, is often done to address CC, but this approach is not unique.^4^^,^^21^^,^^22^^,^^29^ Recently ADMs have been adopted in cosmetic revision breast surgery to provide soft tissue based on the benefits of these matrices in breast reconstructive surgery.^28^ Wagner et al. stated that surgical capsulotomy, replacement of the implant covered with ADM, is an effective method for capsular contraction treatment after breast augmentation^29^, ^30^, ^31^, ^32^

In 2009, Maxwell et al. reported their initial experience using different types of ADM for revision surgery in 78 consecutive cases with breast augmentation in 2 years and demonstrated that ADM can provide soft tissue reinforcement, which is often lacking in patients undergoing revision surgery, improve lower pole support, stabilize the pectoral pocket, and minimize the frequency of recurring CCs (1.6%).^3^^,^^6^^,^^28^ It was one of the biggest series. Concerning primary cosmetic breast surgery, recent publications promote the insertion of ADM at the time of primary breast augmentation to prevent CC, especially in patients with poor quality soft tissue support^13^^,^^33^

Hester et al.^30^ were the first plastic surgeons to insert ADM prophylactically in primary breast augmentation patients, reporting zero CCs in 49 women^11^

Nowadays, we must consider the potential benefits of ADM products against the risk of known complications and disadvantages of ADM. Insertion of a second avascular material adjacent to the breast implant is not without risk: infections and seromas incidence can be higher with their use. These complications must be weighed against the benefit in reducing the risk of CC. Higher expense for the patient, the extra operative time (approximately 30 minutes), and the need for a longer incision, (at least 10 cm) to provide adequate exposure, are other disadvantages of using ADM. Furthermore, it should be noted the minimal risk of transmitting an infectious disease, such as human immunodeficiency virus or hepatitis, or, albeit rare, there is a potential risk of Creutzfeldt–Jakob disease.^33^

## Conclusion

The current evidence from the published scientific literature, albeit limited, suggests the indication for the use of ADM in revision surgery, to support the prosthetic pocket, to minimize the risk of capsular contracture and its recurrence.
